# Electronic and Structural Relaxation of Photoexcited WO_3_ Observed by Femtosecond Resonant X‑ray Emission Spectra

**DOI:** 10.1021/acs.jpclett.5c01062

**Published:** 2025-06-10

**Authors:** Yohei Uemura, Kohei Yamamoto, Yasuhiro Niwa, Thomas Buttiens, Hebatalla Elnaggar, Ru-pan Wang, Masoud Lazemi, Frank de Groot, Tetsuo Katayama, Makina Yabashi, Christopher J. Milne, Toshihiko Yokoyama

**Affiliations:** † FXE Instrument, European X-ray Free Electron Laser Facility, GmbH Holzkoppel 4, 22869 Schenefeld, Germany; ‡ Materials Molecular Science, Electronic Structure, 88301 Institute for Molecular Science, Myodaiji-cho, Okazaki 444-8585, Japan; § Photon Factory, Institute for Materials Structure Science, High Energy Accelerator Research Organisation (KEK), Oho 1-1, Tsukuba 305-0801, Japan; ∥ Institut de minéralogie, de physique des matériaux et de cosmochimie, Sorbonne Université, 4 Pl. Jussieu, 75005 Paris, France; ⊥ FLASH, Deutsches Elektronen-Synchrotron DESY, 22607 Hamburg, Germany; # Materials Chemistry and Catalysis, Debye Institute for Nanomaterials Science, 8125 Utrecht University, Universiteitsweg 99, 3584 CA Utrecht, The Netherlands; g JASRI, Kouto, Sayo-cho, Hyogo 679-5198, Japan; h RIKEN SPring-8 Center, Kouto, Sayo-cho, Hyogo 679-5198, Japan

## Abstract

Photoexcited states of tungsten trioxide (WO_3_) were observed using femtosecond high-energy-resolution fluorescence detection X-ray Absorption Spectra (HERFD-XAS) and resonant X-ray emission spectra (RXES). In the initial state of the photoexcitation (∼100 fs), the W L_3_ edge XAS shifts to lower energy and the energy levels of the *t*_2g_ and *e*_g_ peaks are modulated due to the photoexcited electrons in the conduction band. The electronic state of the photoexcited W atoms is modified by 500 fs. The crystal field splitting between the *t*_2g_ and *e*_g_ peaks shrinks by 500 fs, which indicates local structural changes around the W atoms due to the formation of polarons. HERFD-XAS and RXES provide more details about the early state of the photoexcited states of WO_3_. This work demonstrates that the detailed dynamics of 5d elements in the femtosecond range can be addressed with HERFD-XAS/RXES.

## Graphical abstract

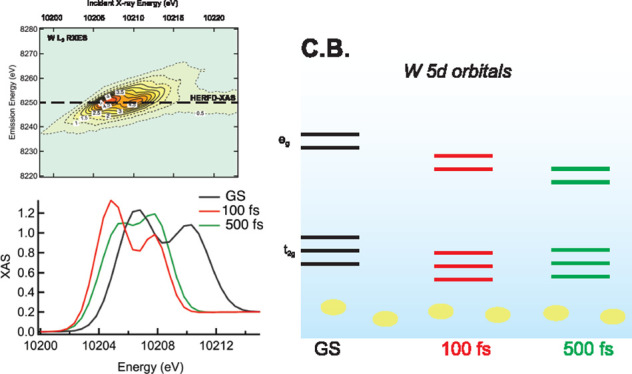

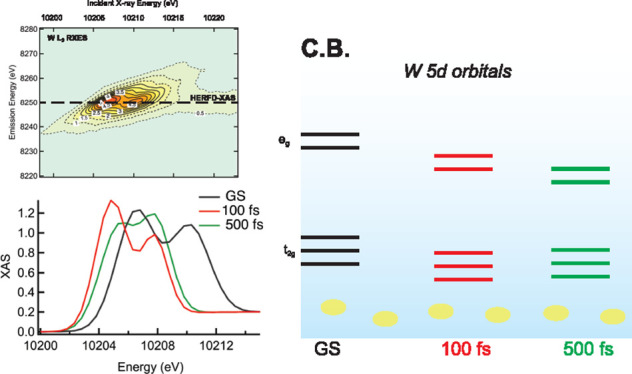

The solar-assisted photochemical water splitting (WS) reaction is an ideal chemical process to produce hydrogen, and photocatalysts and photoelectrodes assist the WS reaction by absorbing the solar spectrum and providing electrons and holes that primarily promote the WS reaction.^1–4^ Tungsten trioxide (WO_3_) is one of the well-studied photocatalysts for the WS reaction.^5,6^ WO_3_ is a wide-bandgap semiconductor, and its bandgap is greater than 2,6 eV, which allows WO_3_ to absorb visible light. Although the energy level of the conduction band of WO_3_ is not high enough to promote hydrogen production from water, the top of the valence band is significantly lower than the electrochemical potential of the oxidation of water^3,6^ to promote oxygen evolution, which is the rate-limiting step of the WS reaction. Combining WO_3_ with another photocatalyst that is active for hydrogen production, such as tantalum oxynitride (TaON), forms an efficient photocatalytic system for the WS reaction (a Z-scheme photocatalyst).^5,6^

The photocarrier properties and dynamics of WO_3_ are essential to understand the photocatalytic properties of WO_3_ and to develop new catalysts that promote the WS reaction more efficiently. Photocarrier dynamics in WO_3_ has been studied using optical pump–probe spectroscopy^7–12^ and THz spectroscopy.^13^ These studies demonstrate charge-carrier dynamics in time domains ranging from femtoseconds to microseconds. Although optical spectroscopies are widely employed to study the photoexcited states of photocatalysts, they cannot address changes in the structures and electronic states of photocatalysts directly. X-ray absorption spectroscopy (XAS) has been used to study the structures or electronic states of materials, in particular, heterogeneous catalysts. Since each element has a unique absorption edge, XAS can provide detailed information about a specific element in a sample. Owing to the progress of femtosecond lasers and X-ray facilities such as synchrotrons and X-ray free electron lasers (XFELs), XAS can be used to study photoexcited states of materials combined with a pump–probe methodology in the time domains from femtoseconds to microseconds. Photoexcited states of metal oxides such as TiO_2,_ ^14,15^ ZnO,^16^ a-Fe_2_O_3_ ^17–19^ and WO_3,_ ^20–23^ have been studied using the pump–probe XAS technique. Since XAS is used to demonstrate changes of the electronic states of key elements in photocatalysts, transient XAS provides complementary information to grasp the origin of photocarriers in photocatalysts. We observed the photoexcited dynamics of WO_3_ using the pump–probe XAS methodology at the SPring-8 Angstrom Compact Free Electron LAser (SACLA), which is an XFEL facility in Japan. We found that an intermediate state formed 100 ps after photoexcitation,^22,23^ and an anisotropic structural change occurred with the formation of the intermediate state.^20,22^ These results demonstrate how charge carriers are stabilized in the photoexcited WO_3_, which allows us to understand more details about the photocarriers in WO_3_.

Although we have previously succeeded in measuring the photoexcited states of WO_3_ in the picoseconds range, we could not observe the initial photoexcitation state of WO_3_ because of the limitation of the time resolution due to the timing jitter in the XFEL^21^ and the energy resolution of W L_3_ edge XAS due to the core-hole lifetime broadening. The limitations in time and energy hinder further exploration of the early stage of the photoexcited states of WO_3_, which is crucial for understanding how the metastable state is formed after photoexcitation. Beyond the limitations, we employed the arrival timing monitor (ATM) and High Energy Resolution Fluorescence Detection XAS (HERFD-XAS), which improve the time resolution and the energy resolution of W L_3_ XAS, respectively. Pump–probe HERFD-XAS was proposed to potentially illustrate more details about the electronic state or structural changes of photoexcited states, in particular, femtosecond regimes. However, there has been only one example of utilizing HERFD-XAS to investigate photoexcited states.^24^ Since HERFD-XAS is a photon-in-photon-out measurement, it requires a long time to obtain HERFD-XAS with a decent signal-to-noise ratio (S/N). We demonstrate that the pump–probe HERFD-XAS methodology is feasible, and it shines a light on the dynamics of WO_3_.

All of the experiments were performed at BL3 EH2, SACLA. A suspension of WO_3_ nanoparticles was prepared and flowed using a liquid jet system. W L_3_ XAS or resonant X-ray emission spectra (XES) were measured simultaneously. A more detailed description of the experiments is found in the Supporting Information.

Pump–probe W L_3_ XAS spectra are displayed in Figure 1(a). We reported that three energy points display distinctive kinetics denoted as peaks A, B and C, respectively.^23^ Peak A appears once after WO_3_ is photoexcited, while peak B grows gradually after the appearance of peak A. The fast and slow kinetics are seen at peak C. These were observed in a period of tens of picoseconds (<100 ps). The ATM can determine the arrival time of an X-ray pulse and a laser pulse with a precision of 4 fs.^25^ The actual delay for each X-ray pulse is corrected based on the data from the ATM. To enhance the measurement efficiency, we employed a feedback system that suppresses the timing jitters/drifts between X-ray pulses and laser pulses based on the data from the ATM. Figure 1(b) displays the kinetic trace at peak A. We successfully observed the fast decay at peak A without the postprocessing of the ATM data.^21^ The instrument response function (IRF) was estimated by the convolution of a step function with a Gaussian function whose full width at half-maximum (fwhm) is 180 fs. We employed a 150-μm liquid jet to flow the sample, and the IRF from the group velocity mismatch is supposed to be ∼150 fs. Since the overall IRF is close to the IRF from the group velocity mismatch, the jet thickness mainly contributed to the IRF. The kinetics at peak A was estimated using a single exponential function, and its decay time was 210 ± 30 fs. This indicates that the initial excited state decays within 500 fs after photoexcitation. Although the fast kinetics were successfully observed, it was still hard to identify the photoexcited state of WO_3_ using fluorescence yield (FY) XAS due to the core-hole lifetime broadening. The core-hole lifetime broadening makes the energy resolution of XAS worse, and some key features of XAS can be smeared out. For example, a tungsten atom in WO_3_ is surrounded by six oxygen atoms, and the 5d orbitals of the tungsten atom are split into different energy levels. In theory, W L_3_ XAS should reflect this split of the 5d orbitals, i.e., two peaks corresponding to 2p → 5d­(*t*_2g_) and 2p → 5d­(*e*_g_), respectively, should be observed. However, such features cannot be seen if XAS is measured by a FY mode, as shown in Figure 1(a). Instead of collecting X-ray fluorescence using a photodiode, resonant X-ray emissions (RXES) with scanning the incident X-ray energy improves the energy resolution of XAS, known as HERFD-XAS. While a photodiode collects all emission signals from a sample in the FY mode, a specific emission line within a finite energy range is collected in HERFD-XAS. Limiting the spectral width of the emission line helps to observe XAS ascribed to a specific transition (see Figure S3).^26^ This improved the energy resolution of XAS, and it allowed us to study detailed features smeared out in FY XAS.

**Figure 1:**
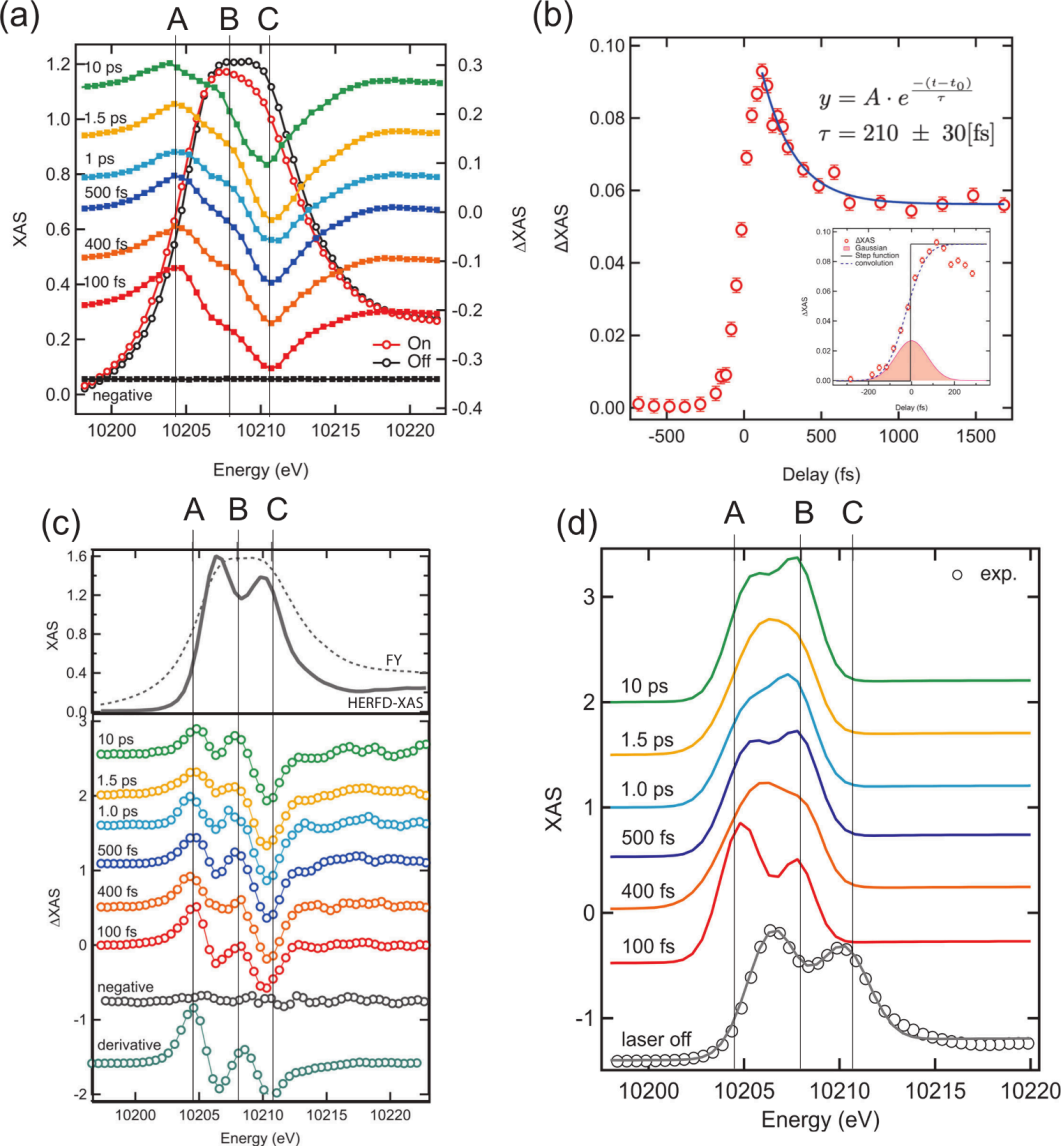
(a) Pump–probe XAS spectra of WO_3_ at different delays. (b) The kinetic trace of XAS at peak A in (a). (c) Pump–probe HERFD-XAS of WO_3_ at the corresponding delays to those in (a). The first derivative of the laser off spectrum is also displayed. (d) shows the reconstructed excited state spectra.

XAS measured by the FY mode and the HERFD mode is displayed in Figure 1(c) (in top panel). The XAS measured by the FY mode looks much broader than the XAS from the HERFD mode. For example, the rise of the X-ray absorption (from the onset to the peak top) is estimated to be 7–8 eV from the FY XAS while it with HERFD-XAS is to be ∼3.5 eV. A, B and C represent the same energy points as displayed in Figure 1(a). In HERFD-XAS, the same energy points as in Figure 1(a) display noticeable changes. At 100 fs, there are two factors considered to induce the spectral change: one is a red shift of the W L_3_ XAS due to additional electrons in the W 5d bands. After photoexcitation, electrons occupy the W 5d band, which form the conduction band. The binding energy of 2p electrons can be decreased by the additional electrons in W 5d orbitals, resulting in the red shift. The features of the difference of HERFD-XAS at 100 fs look like the first derivative of the unpumped HERFD-XAS, which implies that the spectral shift contributes to the change of the HERFD-XAS of the photoexcited state at 100 fs. The other is the change of the energy levels of the W 5d orbitals. In Figure 1(c), two peaks corresponding to *t*_2g_ and *e*_g_ orbitals are observed using HERFD-XAS while they overlap to be a single broad peak in the FY XAS. The peak separation corresponds to the crystal field splitting of the W 5d orbitals. At 100 fs, electrons in the valence band are excited to the conduction band consisting of W 5d orbitals and holes are created in the valence band from O 2p orbitals. This effectively decreases the chemical bonding strength between O 2p and W 5d orbitals, which implies that the antibonding states become less antibonding. The *e*_g_ states have sigma bonding, and the reduced bonding effect is stronger on the *e*_g_ states. As a result, the crystal field splitting can be decreased.

At delays of 400 and 500 fs, the magnitudes of peaks B and C become more prominent while the magnitude of peak A gets smaller. A relaxation process is expected considering the kinetics at peak A. Excited electrons can occupy lower positions in the conduction band. The difference spectrum changes further at 1 or 10 ps. A kinetic process which lasts to a delay of 50 ps was observed in the previous experiments.^22,23^ A metastable state with a structural change emerges in the process. We conclude that the changes at 1 or 10 ps correspond to the formation of a metastable state with a modified structure before the longer-lived state is reached.

To investigate further details of the photoexcited states of WO_3_, we extracted HERFD-XAS of laser-on. Yamazoe et al. reproduced W L_3_ XAS spectra of different W compounds and evaluated the splitting between *t*_2g_ and *e*_g_ peaks.^27^ We employed a method similar to Yamazoe’s to reconstruct the XAS spectra of the photoexcited state of WO_3_ at different delays. First, we used the equation below to reproduce the unpumped HERFD-XASs:

$$\mu \left(E\right)=a\cdot \exp\left(\frac{-{\left(E-E_{t2g}\right)}^{2}}{2\sigma_{t2g}^{2}}\right)+b\cdot \exp\left(\frac{-{\left(E-E_{eg}\right)}^{2}}{2\sigma_{eg}^{2}}\right)+c\cdot \arctan\left(\frac{-\left(E-E_{0}\right)}{\gamma }\right)$$ 1

where the first and second Gaussians stand for the *t*_2g_ and *e*_g_ peaks, respectively, and the third term represents the edge step of W L_3_ HERFD-XAS. This equation successfully reproduced the unpumped HERFD-XAS as shown in Figure 1(d). Each difference HERFD-XAS is described as follows:

$$\Delta \mu \left(E\right)=\alpha \left(\mu^{*}\left(E\right)-\mu \left(E\right)\right)$$ 2

where α stands for the excitation coefficient and μ*­(*E*) for HERFD-XAS of the excited state. The same equation as that shown above was used to reproduce μ*­(*E*) and μ­(*E*). When Δμ­(*E*) was reproduced, the parameters were fixed for μ­(*E*).

The reconstructed HERFD-XAS results are shown in Figure 1(d). The reconstructed spectra elucidate how the spectral change of the excited state affects the kinetic trace in Figure 1(b) and the features of the difference spectra in Figure 1(c). The reconstructed spectra appear at lower energy, as expected from the difference spectra. At 100 fs (right after the photoexcitation), the spectrum shifts by −1.6 eV and the *t*_2g_ peak appears around peak A, consistent with the fast rise shown in Figure 1(b). The splitting of the two peaks decreases by 0.6 eV as mentioned above. In later delays (>400 fs), the spectrum shifts back by +0.5 eV compared to the spectrum at 100 fs. As a result, the XAS intensity at peak A decreases, as seen in Figure 1(b). The second peak of the L_3_ HERFD-XAS of the excited state appears around peak B. The second peak intensity slightly decreases compared to the *e*_g_ peak of the ground state. In addition, the gap between the first and second peaks shrinks by ∼1 eV compared to the energy splitting between the *t*_2g_ peak and *e*_g_ peak of the ground state. The shrinking of the peak splitting implies the distortion of the local structure of photoexcited W atoms.

It should be noted that the ratio of *t*_2g_ and *e*_g_ peaks varies after the photoexcitation. The ratio of the areas of the two peaks is estimated to be ∼0.75 for the ground state and the ratio is to be 0.5 at 100 fs. The ratio becomes larger along the delays. From multiplet calculations of W L_3_ HERFD-XAS, this trend is reproduced by changing the spin–orbit coupling term (Figure S4) The HERFD-XAS in the ground state, at 100 and 400 fs are reproduced well if the spin–orbit coupling term is set to 0. On the other hand, HERFD-XAS at 500 fs, 1 ps, and 10 ps are reproduced by setting the spin–orbit coupling term to 1 although HERFD-XAS at 1.5 ps is reproduced by the spin–orbit coupling term of 0. Since the W 5d orbitals are delocalized when WO_3_ is in the ground state, the spin–orbit coupling term can be set to 0. On the other hand, it is necessary to set the spin–orbit coupling term to a finite value to reproduce HERFD-XAS of the photoexcited states. Since the degree of the spin–orbit coupling reflects the localization of valence orbitals,^28^ photoexcited W atoms could be more localized. For instance, the local structure of the photoexcited W atoms is distorted after photoexcitation, and the W atoms are more “isolated” due to the structural change, where the contribution of spin–orbit coupling to W L_3_ XAS becomes significant. Although this should be investigated further, it may reflect the formation of polarons around photoexcited W atoms. Polaron formation in WO_3–*x*_ was observed by Schirmer et al.^29^ (*x* ≥ 0.0001) at low temperatures, and Tao et al. investigated the polaron formation in WO_3_ using hybrid density functional theory calculations.^30^ Although these studies do not directly support the polaron formation upon photoexcitation, we assume that localized charge carriers can be formed after photoexcitation in WO_3_.

2D maps of the difference of RXES at the delays corresponding to those in Figure 1 are shown in Figure 2. As shown in the Supporting Information, the transition of 2p → *t*_2g_ and 2p → *e*_g_ peaks are seen as the diagonal lines (Figure S3). The following scheme emits W L_α_ line:

$$\left|2p^{6}3d^{10}5d^{0}\right\rangle \overset{\mathrm{excitation}}{\to }\left|2p^{5}3d^{10}5d^{1}\right\rangle \overset{\mathrm{emission}}{\to }\left|2p^{6}3d^{9}5d^{1}\right\rangle$$ 3

|2p^6^3d^10^5d^0^⟩ stands for the ground state, |2p^5^3d^10^5d^1^⟩ for the intermediate state and |2p^6^3d^9^5d^0^⟩ for the final state. Since the energy difference between the initial state and the final state is constant, an emission from the same final state appears as a diagonal line. Since we only see a diagonal feature in RXES, the multiplet effects between 2p/3d and 5d orbitals are negligible. Therefore, the difference in XES in Figure 2 indicates how *t*_2g_ and *e*_g_ states evolve. At 100 fs, the positive feature and negative feature appear. The positive feature is seen at a lower energy position compared to the *t*_2g_ state of the ground state while the negative feature is seen at the energy position close to the energy position of *e*_g_ state of the ground state. This is because the difference in XES is attributed to the spectral shift and the shifts of the energy levels between the *t*_2g_ and *e*_g_ states at 100 fs. Positive and negative features between the *t*_2g_ and *e*_g_ states are not seen clearly. The XES intensity of the photoexcited state in the region can be relatively close to the intensity of the *e*_g_ ground state. The increase or decrease of the RXES is not clearly seen compared to outside the two states. In later delays, a rising feature appears between *t*_2g_ and *e*_g_, which is indicated by an arrow in Figure 2(b),(c). This indicates that new bound states appear. At 10 ps, the new bound state is more pronounced. This feature is seen in Figure 1(c) as peak B. It should be noted that these features cannot be observed if a standard FY XAS is employed. In FY XAS, the signal at each energy point is the sum of emission signals detected in a photodiode. FY XAS is identical to the projection along the emission energy in Figure 2. For example, a difference of XES at 10208 eV at 400 and 500 fs has positive and negative features, and they cancel out their features with each other. Therefore, the feature seen in HERFD-XAS (Figure 1(c)) cannot be noticed.

**Figure 2:**
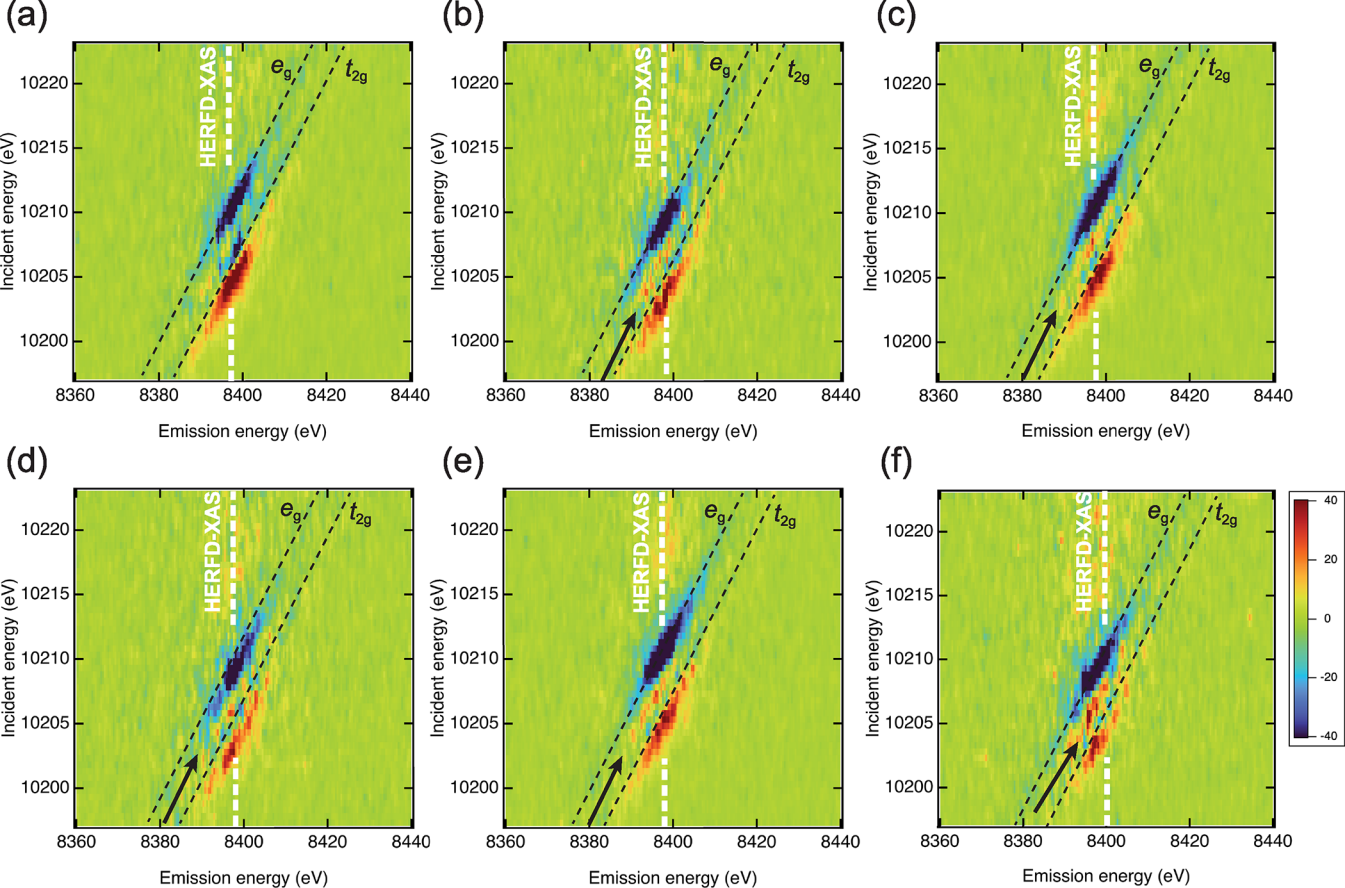
Pump–probe RXES at different delays: (a) 100 fs, (b) 400 fs, (c) 500 fs, (d) 1 ps, (e) 1.5 ps, and (f) 10 ps. The white dash line indicates the energy to form HERFD-XAS in Figure 1(c).

Figure 3 summarizes the mechanism of the photoexcitation of WO_3_. After photoexcitation (<180 fs, Figure 3(b)), WO_3_ absorbs the 400 nm laser (<100 fs), electrons in the valence band, mainly consisting of O 2p orbitals, are excited to the conduction band consisting of W 5d orbitals. Due to the shielding effect from the excited electrons, the binding energy of W 2p electrons becomes smaller, and the photoexcited W L_3_ XAS shifts to a lower energy. W L_3_ XAS shifts toward a lower incident energy; i.e., a red shift occurs. The spin–orbit coupling term is set to 0 to reproduce HERFD-XAS at 100 fs, which reflects the W 5d orbitals are delocalized. Therefore, we suppose that the excited electrons in the W 5d orbitals are delocalized. In addition to the red shift of the XAS spectrum, the energy positions of *t*_2g_ and *e*_g_ peaks shift, and the energy gap between the two states becomes smaller. The excited electrons occupy the conduction band consisting of *t*_2g_ and *e*_g_ orbitals, corresponding to the antibonding states of W–O, as shown in Figure S5 in the Supporting Information. If the antibonding states are occupied, then the W–O bonds can be weaker. The gap between *t*_2g_ and *e*_g_ peaks reflects the strength of the crystal field. As the W–O bond becomes stronger, the crystal field splitting becomes larger. On the other hand, the crystal field splitting becomes smaller if the W–O bonds get weaker. Since the energy gap between *t*_2g_ and *e*_g_ peaks is decreased, the W–O bonds should be weaker than in the ground state. Since the lower part of the conduction band, where the electrons in the valence band are excited by the pump laser, is formed by *t*_2g_ orbitals. The *t*_2g_ orbitals have π symmetry; i.e., the orbitals are distributed between two oxygen atoms, as shown in Figure S5. At 100 fs, not a specific W–O becomes weaker, but all the W–O bonds get weaker due to the excited electrons.

**Figure 3:**
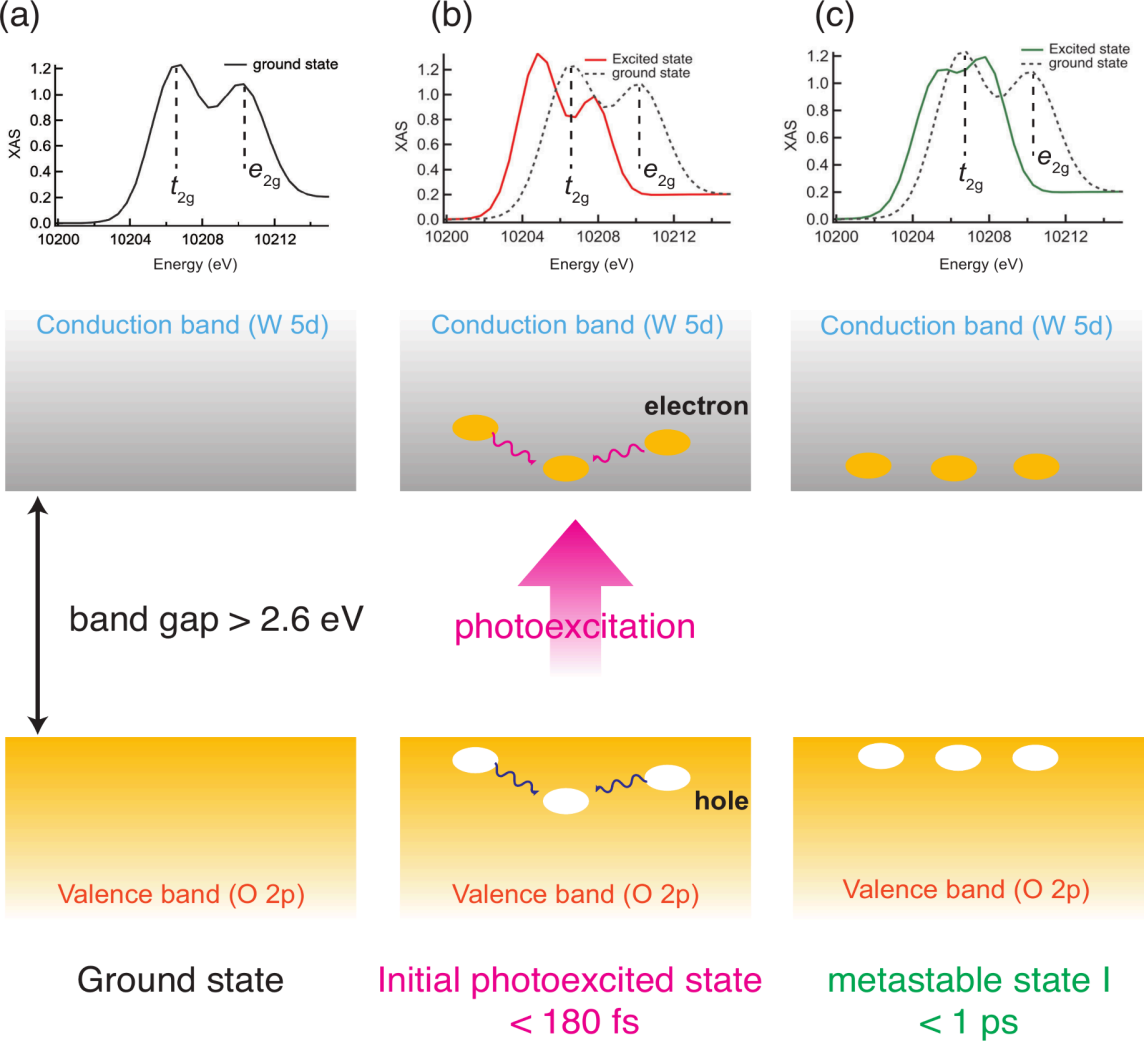
A sketch of proposed photoexcitation and relaxation mechanism: (a) optical ground state, (b) the initial photoexcited state, (c) metastable state. After photoexcitation (the initial photoexcitation state shown in Figure 3(b)), electrons in the conduction band are delocalized. The HERFD-XAS shifts in lower energies due to the electrons in the conduction band. In later delays shown in Figure 3(c), the electrons are localized, which is supported by the change of the spin–orbit coupling term for the multiplet calculations of W L_3_ HERFD-XAS.

A fast decay at peak A is observed below 500 fs, as shown in Figure 1(b) to form the metastable state (Figure 3(c)). The fast decay process observed at Position A in Figure 1(a) is attributed to the blue shift of the photoexcited W L_3_ XAS and the splitting between *t*_2g_ and *e*_g_ orbitals. Lee et al. observed the photoexcitation state of WO_3_ using a pump–probe near-infrared spectroscopy,^8^ and they found a fast decay process that occurs below 1 ps. They assigned this process to a hot-carrier relaxation process due to hot-carrier scattering, since they did not observe features from electron–phonon coupling in the NIR spectra. In our observations, a similar scenario can be considered: the excited electrons are distributed in the conduction band and occupy different energy levels since the energy of the pump laser (3.1 eV) is larger than the band gap of WO_3_ (∼2.6 eV). These hot carriers collide with each other and transfer energies. The hot carriers reach equilibrium through scattering and occupy lower energy positions in the conduction band. Besides the electron relaxation, the splitting between the *t*_2g_ and e_g_ peaks becomes smaller by 1 eV. The change of the crystal field splitting at sub-ps time scale indicates the change in the hybridization of W and O orbitals. The W 5d orbitals and the O 2p orbitals have less interaction compared to the ground state due to the decrease of the charge difference. It can be expected that the W–O bonds are slightly elongated along the bond direction, which affects more the *e*_g_ orbitals, of σ symmetry, i.e. the energy level of *e*_g_ goes down. Indeed, we observed a small change on the pre-edge peak of W L_1_ XAS, which is sensitive to the local symmetry of W atoms, in the shorter delays, although the main change comes from the red shift of the L_1_ spectrum.^22^ The change in the W L_1_ edge in XAS indicates that the octahedral unit in WO_3_ could be distorted. We expect that the excited electrons are localized around W atoms to form a polaron state.

We observed the photoexcited states of WO_3_ using HERFD-XAS/RXES at SACLA. After photoexcitation (100 fs), the electronic states of the photoexcited W atoms are modulated, which causes the spectral shift of the W L_3_ XAS, and modulate the energy positions of the *t*_2g_ and e_g_ peaks. In addition, the modulation of the electronic states results in shrinking the energy split between the *t*_2g_ and *e*_g_ peaks. By 500 fs, the splitting of the *t*_2g_ and e_g_ peaks shrinks further. We assume that electrons are localized around W atoms around 500 fs accompanied by structural change to form a polaron state. HERFD-XAS/RXES changes further, corresponding to the structural change around the photoexcited W atoms we proposed.^20,22^ Measuring pump–probe HERFD-XAS/RXES of WO_3_ was feasible and provided more detailed information about the dynamics of WO_3_ even though the repetition rate of XFEL is not so high. This opens up pump–probe HERFD-XAS for other energy conversion materials based on 5d elements such as Ta, Ir, Pt. Using XFELs with a high repetition rate such as LCLS-II^31^ or European XFEL^32^ enhance the capabilities of pump–probe HERFD-XAS/RXES, which leads to understanding the mechanisms of photocatalysts/photoelectrodes further.

## Supplementary Material



## Notes

Experimental details, resonant X-ray emission spectrum (RXES) of WO_3_, multiplet calculations of W L_3_ HERFD-XAS, orbital energies and contributions in WO_3_ (PDF)

## $

NanoTerasu Center, National Institutes for Quantum Science and Technology NanoTerasu, 468-1, Aoba, Aramaki, Aoba-ku, Sendai, Miyagi, Japan
