# Supplementary material for: Electronic and Structural Relaxation of Photoexcited WO3 Observed by Femtosecond Resonant X‑ray Emission Spectra
Source: J Phys Chem Lett. 2025 Jun 10;16(24):6138–45. doi: 10.1021/acs.jpclett.5c01062 (PMC12183732; doi:10.1021/acs.jpclett.5c01062)
Supplement: Supplementary file 1 [file jz5c01062_si_001.pdf]

Supporting Information

Electronic and structural relaxation of Photoexcited WO_3 Observed by Femtosecond Resonant X-ray Emission Spectra

Yohei Uemura[a], Kohei Yamamoto[b$], Yasuhiro Niwa[c], Thomas Buttiens[b$$], Hebatalla Elnaggar[d], Ru-pan Wang[e], Masoud Lazemi[f], Frank de Groot[f], Tetsuo Katayama[g,h], Makina Yabashi[h], Christopher J. Milne[a], Toshihiko Yokoyama[b]*

a FXE Instrument, European X-ray Free Electron Laser Facility GmbH

Holzkoppel 4, 22869 Schenefeld, Germany

E-mail: yohei.uemura@xfel.eu

b Materials Molecular Science, Electronic Structure, Institute for Molecular Science

Myodaiji-cho, Okazaki 444-8585, Japan

c Photon Factory, Institute for Materials Structure Science

High Energy Accelerator Research Organisation (KEK)

Oho 1-1 Tsukuba 305-0801, Japan

d Institut de minéralogie, de physique des matériaux et de cosmochimie,

Sorbonne Université

4 Pl. Jussieu, 75005 Paris, France

e FLASH, Deutsches Elektronen-Synchrotron DESY

Deutsches Elektronen-Synchrotron DESY, 22607 Hamburg, Germany

f Materials Chemistry and Catalysis, Debye Institute for Nanomaterials Science, Utrecht University,

Universiteitsweg 99, 3584 CA, Utrecht

g JASRI,

Kouto, Sayo-cho, Hyogo 679-5198, Japan

h RIKEN SPring-8 Center

Kouto, Sayo-cho, Hyogo 679-5198, Japan

$Current address: NanoTerasu center, National Institutes for Quantum Science and Technology NanoTerasu, 468-1, Aoba, Aramaki, Aoba-ku, Sendai, Miyagi, Japan

Table of Contents

Experiments.. 2

Resonant X-ray Emission Spectrum (RXES) of WO_3 4

Multiplet calculations of W L_3 HERFD-XAS................................. 5

Orbital energies and contributions in WO_3................................ 7

References 7

Experiments

The pump-probe XAS and RXES measurements were performed at BL3 EH2. To measure XAS, photodiodes were employed. To detect the incident x-ray intensity, a kapton film was used as a scatter and the scattered x-ray was collected by two photodiodes. To measure the fluorescecne x-ray intensity, a photodiode was placed close to the x-ray spot on the sample. A photodiode from Hamamatsu photonics was placed next to the sample (the photosensitive are is 10 mm x 10 mm, its cutoff frequency is 40 MHz). The distance between the sample and the fluorescence detector was 10 mm (the solid angle of the detector is 10). To obtain x-ray emission spectra, 6 von Hamos analysers were employed and a Multi-port CCD detector (MPCCD) was used to collect XES spectra. The Si(444) reflection was used to obtain XES spectra. The energy resolution of W $L_{\alpha1}$ line was estimated as 0.6 eV. Two Si(111) channel-cut crystals were used to mochromatise the x-ray. The energy resolution of the incident x-ray was ~ 1 eV. The ovarall energy resolution of RXES was 1.1 – 1.2 eV. A 150-µm jet was used and the sample was always circulated using a peristaltic pump. The speed of the jet flow was set to 10 ml/min, which was fast enough to replace the WO_3 nanoparticles in the solution around the x-ray spot. The flow was monitored by a microscope, and it was very stable while the XAS measurements were performed. The sample was the suspension of WO_3 nanoparticles (solvent: water). The source nanosuspension was purchased from GS Alliance Co. Ltd. (the content of

WO_3 was 5 wt%). The source suspension was diluted using distilled water to prepare a 10 mM WO_3 suspension. The X-ray diffraction pattern of the nanoparticles is shown in Fig. S2. The XRD was measured using a Debye-Scherrer geometry (Brucker D8 Advance, X-ray source: Cu Kα_1). The dried WO_3 nanoparticles and the suspention were filled into a glass capillary. To measure the diffraction x-rays of the dried sample efficiently, the sample was diluted using Cellulose. The background was subtracted using DIFFRAC.EVA (ver 4.3). A broad peak seen around 22.5 degrees comes from the XRD of Cellulose in the dried sample. The structure of

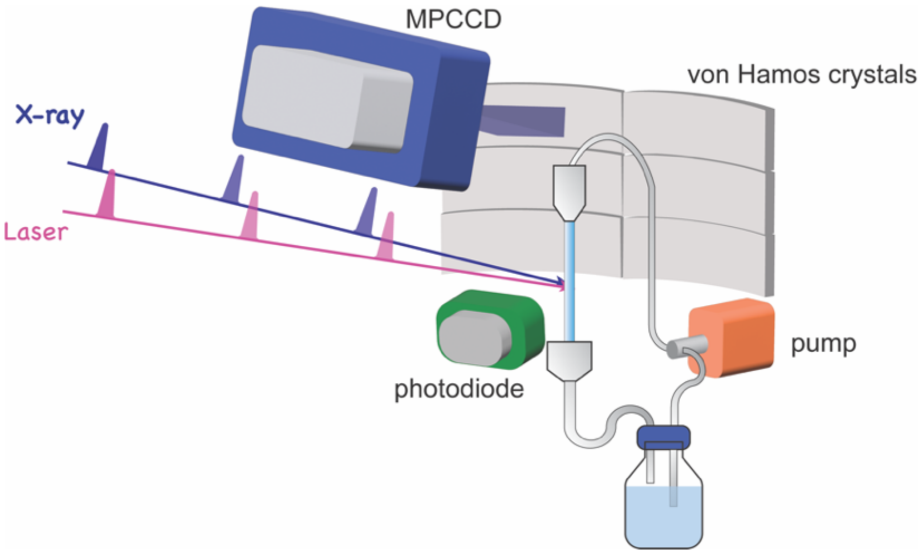

Fig. S1 A schematic illustration of the experimental setup at BL3 EH2, SACLA.

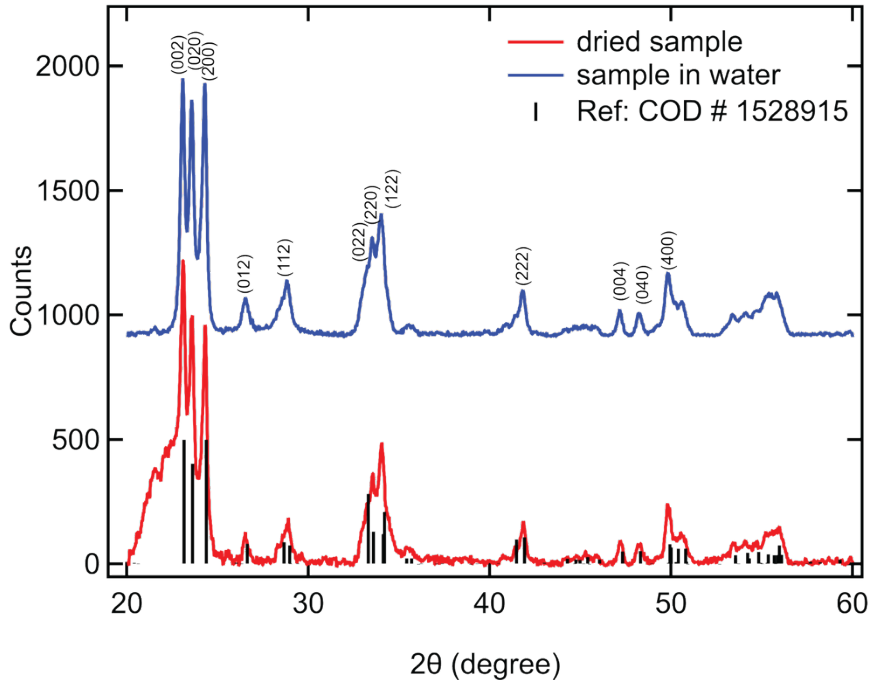

Fig. S2 X-ray diffraction pattern of WO_3 nanoparticles

WO_3 is confirmed to be a monoclinic phase[1] and the average particle size was estimated to be ~30 nm from the diffraction pattern.

There is intringic jitters between x-ray pulses and laser pulses which translates into the fluctuations of the timing between x-rays and laser pulses. In order to minimise the timing jitters between x-rays and laser pulses, we employed a feedback system based on the arrival timing monitor developed in SACLA. The feedback system was able to reduce the timing drifts and jitters between x-rays and laser pulses. In the past, we needed to analyse the arrival timing monitor to follow fast kinetics which can be smered out by the timing jitters. Owing to the feedback system, we observed a fast decay process without analysing the data from the arrival timing monitor. The feedback system allowed us to measure XAS and RXES more efficiently.

Resonant X-ray Emission Spectrum (RXES) of WO_3

A static RXES of WO_3 is displayed in Fig. S3 (a). When RXES is measured, the emission energy from a specific final state can vary over the scan range because of the energy conservation among the incident x-ray, the emitted x-ray and the energy difference between the initial and the final state (Fig. S3(b)). Therefore, the x-ray emission from an identical final state appears as a diagonal line. In Fig. S3(a), there are two diagonal features seen, which are transitions to t_{2g} and e_g orbitals, respectively. Since there are no additional features observed, 2p5d and 3d5d multiplet effects are very small. Therefore, we consider that core holes in 2p and 3d orbitals are localised, and an electron in 5d orbitals is delocalised. RXES of W L_α line displays the changes in the electronic states of t_{2g} and e_g orbitals.

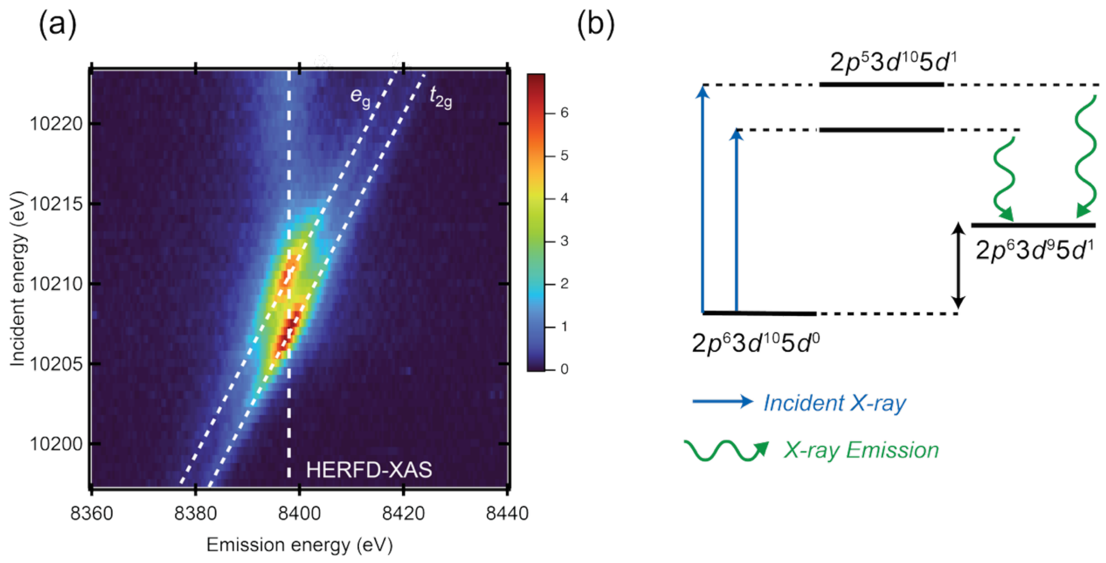

Fig. S3 (a) static RXES of WO_3 nanoparticles (b) a schematic of RXES of W.

Multiplet calculations of W L_3 HERFD-XAS

Multiplet calculations of W L_3 HERFD-XAS were performed using Quanty [2]. These calculations take into account:

(i) The intra-atomic Coulomb interaction (direct/exchange), which has been scaled to 20% of its atomic value due to the nephelauxetic effect for all calculation.

(ii) The crystal field splitting which is considered to be O_h for W in WO_3. The value of the crystal field splitting,10Dq, was determined by comparing the calculations to the experimental spectra.

(iii) The core and valence spin-orbit coupling (SOC). The core SOC was kept constant to its atomic value. The valence SOC was determined by comparing the calculations to the experimental spectra.

The results of the calculation are shown in Fig. S4 where the experimental HERFD-XAS measured at different delay times are plotted in the left column, the corresponding calculations in the middle column, and the experimental and calculation pump-probe HERFD-XAS difference spectra in the right column.

The ground unpumped state can be well reproduced with the multiplet calculations of W^{6+} with a 10Dq splitting of 3.7 eV and completely quenched valence SOC. Two peaks are observed which can be assigned to transitions into the t_{2g} and e_g orbitals. The quenching of the valence SOC indicates that the W 5d orbitals are delocalised. [3] After the pump, at early time delays (100 - 400 fs), the excited state can be well reproduced by calculating W^{5+} with a reduced 10Dq splitting and quenched valence SOC. From 500 fs, the ratio of the t_{2g} and e_g peaks is reversed. This reversal of the peaks' ratio is well reproduced by taking into account the valence SOC. The need to include the valence SOC reflects that electrons in the valence band start to partially localize. [3] We interpret this localization as a signature of polaron formation. We note that the spectrum at 1.5 ps time delay falls out of trend as the valence SOC has to be quenched to reproduce the experimental data. We suspect that there might be a problem with the timing for this delay point.

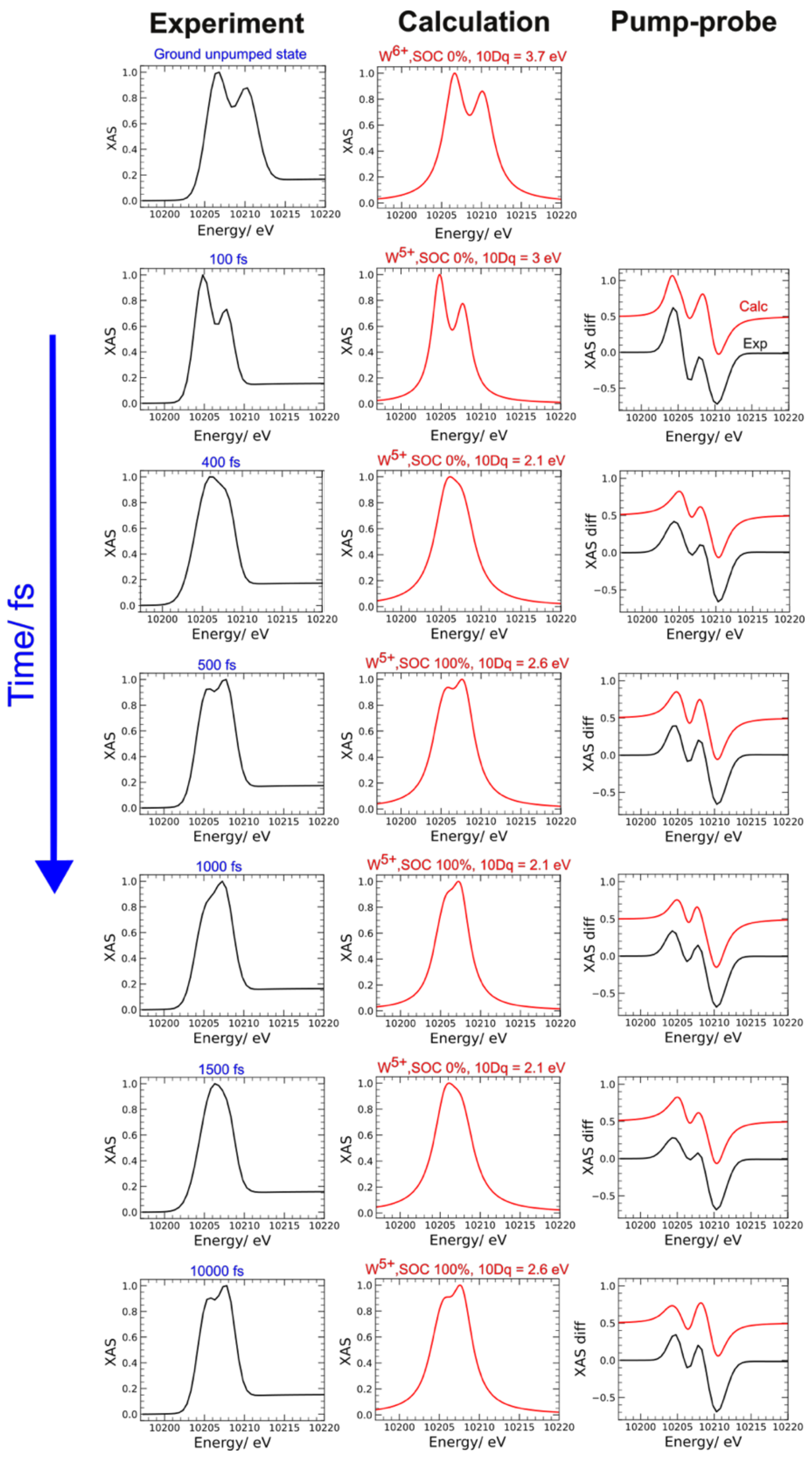

Fig. S4 The multiplet calculations for W L_3 HERFD-XAS: Exp. stands for the reconstructed spectrum of the excited state and the relevant calculation parameters are reported in the title of the subplots.

Orbital energies and contributions in WO_3

Orbital energies and contributions to the band structure of WO_3 are described in Figure S5 referring Goodenough's diagram[4]. t_{2g} orbitals contribute to the lower part of the conduction band of WO_3 and e_g orbitals contribute to the higher part of the conduction band. t_{2g} orbitals have a π symmetry whereas e_g orbitals have a σ symmetry. t_{2g} orbitals are distributed between two oxygen atoms while e_g orbitals are distributed in the direction of W-O bonds.

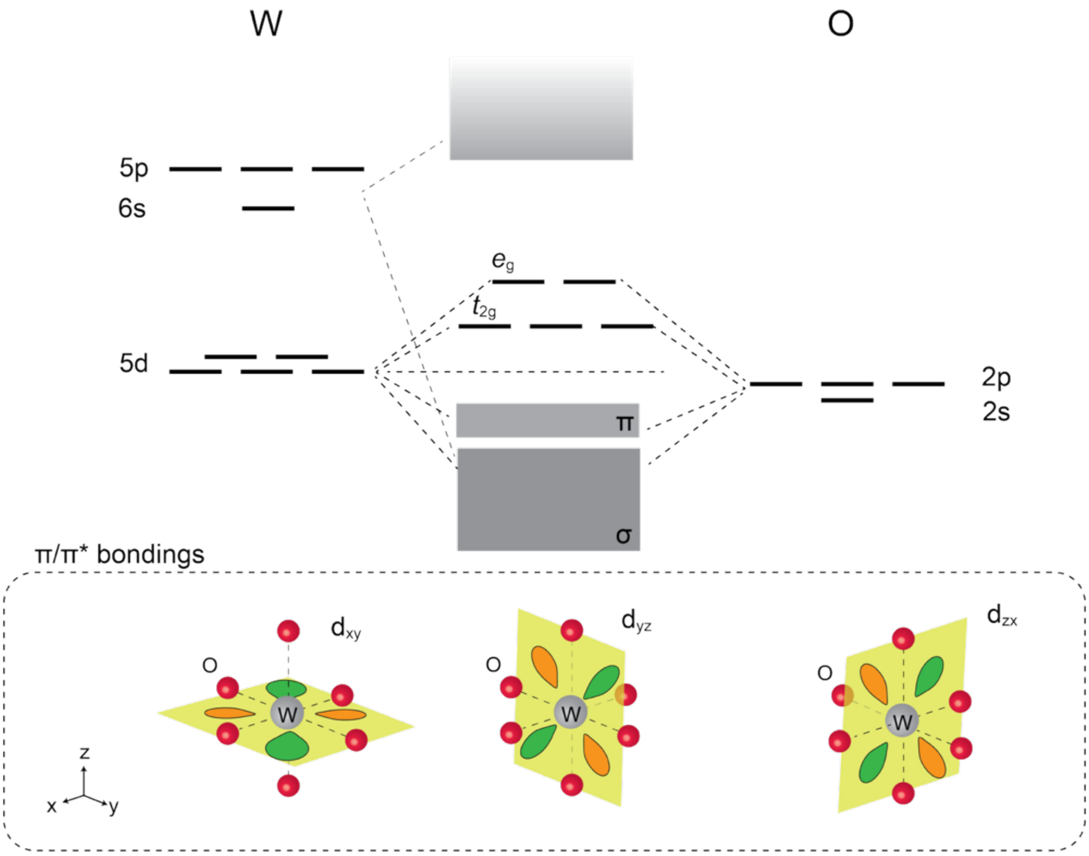

Fig. S5 Schematic for orbital energies of WO_3 and π or π* bonds.

References

[1] P. J. Boruah, R. R. Khanikar, H. Bailung, *Plasma Chem. Plasma Process.* **2020**, *40*, 1019-1036.

[2] M. W. Haverkort, *Journal of Physics: Conference Series* **2016**, *712*, 012001.

[3] (a)F. M. F. de Groot, *Physica B: Condensed Matter* **1995**, *208-209*, 15-18; (b)F. M. F. de Groot, Z. W. Hu, M. F. Lopez, G. Kaindl, F. Guillot, M. Tronc, *J. Chem. Phys.* **1994**, *101*, 6570-6576.

[4] J. B. Goodenough, *Czechoslovak Journal of Physics B* **1967**, *17*, 304-336.
